# Supplementary figures and images for: Major bleeding risk and mortality associated with antiplatelet drugs in real-world clinical practice. A prospective cohort study
Source: PLoS One. 2020 Aug 7;15(8):e0237022. doi: 10.1371/journal.pone.0237022 (PMC7413418; doi:10.1371/journal.pone.0237022)

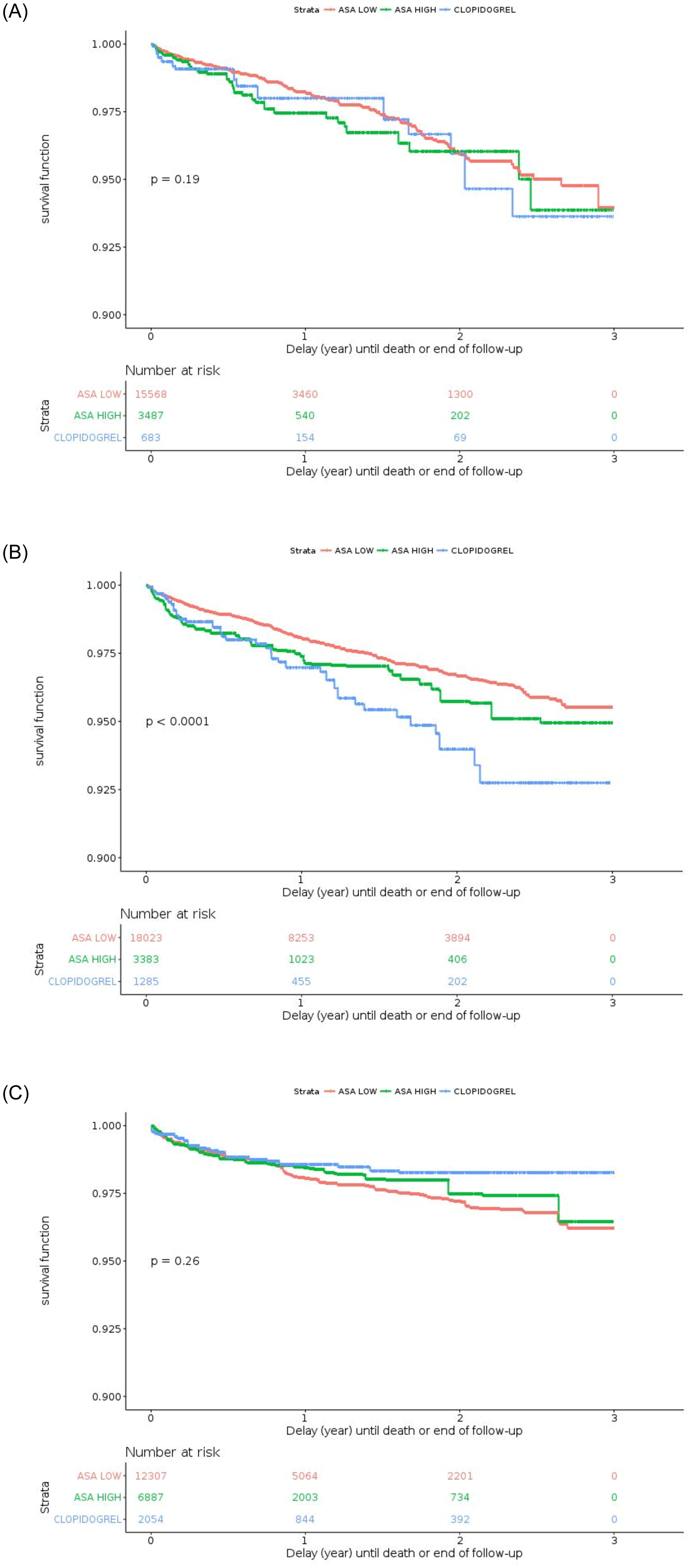

Supplement: S1 Fig — Panel A: Primary prevention without risk factors. Panel B: Primary prevention with risk factors. Panel C: Secondary prevention. (TIF) [file pone.0237022.s001.tif]
