# Supplementary material for: Major bleeding risk and mortality associated with antiplatelet drugs in real-world clinical practice. A prospective cohort study
Source: PLoS One. 2020 Aug 7;15(8):e0237022. doi: 10.1371/journal.pone.0237022 (PMC7413418; doi:10.1371/journal.pone.0237022)
Supplement: S1 File — (DOCX) [file pone.0237022.s002.docx]

**S1 Table. Adapted Charlson Index from Bannay et al.**

| Factors | Weight | ICD-10 code | Medical act code | ATC code |
| --- | --- | --- | --- | --- |
| Congestive heart failure | 2 | I11.0, I13.0, I13.2, I50.x |  |  |
| Peripheral vascular disease | 1 | I70x, I71x, I731, I738, I739, I771, I790, I792, K551, K558, K559, Z958, Z95.9 | EEAFx, ECPFx, ECFAx, EBFAx,, EENFx, EEPFx, EEFAx, EECAx, ENNFx, ENFAx, DGAFx, EDAFx, DGFPx, EDPFx, DGFAx, EDFAx, DGCAx, EDCAx, EDPFx, EDJFx, EDKAx, EDEAx, EDLFx, EDNFx, EDPFx, EDJFx, EDMAx, EANF002, ECNF002, ECJF001, ECCA007, ECCA009, ECCA003, ECCA002, ECMA001, ECKA002, EBNF001, EDEA001, EDLF007, EEJF001, EDCA005, ENAF001, ENAF002, DGKA004, EDNF003, EDKA002 |  |
| Cerebrovascular disease | 1 | G45.x, G46.x, H340, I60x-I69x |  |  |
| Dementia | 2 | F00x–F03x, F051, G30x, G311 |  | N06D * |
| Chronic pulmonary disease | 1 | I278, I279, J40x–J47x, J60x–J67x, J684, J701, J703 |  | R03 § |
| Mild liver disease | 2 | B18x, K700–K703, K709, K713–K715, K717, K73x, K74x, K760, K762–K764, K768, K769, Z944 |  |  |
| Moderate or severe liver disease | 2 | I850, I859, I864, I982, K704, K711, K721, K729, K765, K766, K767 |  |  |
| Hemiplegia | 1 | G041, G114, G801, G802, G81x, G82x, G830–G834, G839 |  |  |
| Moderate or severe renal disease | 2 | I120, I131, N032–N037, N052–N057, N18x, N19x, N250, Z490–Z492, Z940, Z992 |  |  |
| Any malignancy, including leukemia and lymphoma | 3 | C00x–C26x, C30x–C34x, C37x–C41x, C43x, C45x–C58x, C60x–C76x, C81x–C85x, C88x, C90x–C97x |  |  |
| Metastatic solid tumour | 11 | C77x–C80x |  |  |
| AIDS/HIV | 1 | B20x–B22x, B24x, Z21 |  |  |

The different criteria were assessed in the 12 months preceding inclusion date; * at least 3 deliveries; § at least 2 deliveries

**S2 Table. Definition of presumed indication of oral anticoagulant, co-morbidities (according to International Classification of Diseases, 10th revision (ICD-10) or medical act classification) and co-medications (Anatomical Therapeutic Chemical (ATC) system).**

| **Conditions** | **ICD-10 code** | **Medical act code** | **ATC code** |
| --- | --- | --- | --- |
| Coronary diseases | I20xx, I21xx, I22xx, I23xx, I24xx,I255,I256 I258, I259 | DAQM%,DDAA%,DDAF%,DDFF%,DDMA%,DDPF%,DDQH%,DKRP%,DZQM%,EQRP% |  |
| Carotid disease | I65% | EBAA%,EBCA%,EBFA%,EBQJ%,EBQM%,ECFA% |  |
| PVD | I70%, I73%, I771, I792,Z9580, K550, R02, K558,K559,N280, I74%,M352 | NZQN%,NZQJ%,MZQN%,EZQM%,EZPA%,EZMH%,EZAF%,ENNF%,ENFF%,ENFA%,ENAF%,EMQJ%,EMQH%,ELQM%,  ELQH%,EKQH%,EEPF%,EENF%,EEJF%,EEGA%,EEFA%,EECA%,EEAF%,EEAA%,EDQM%,EDPF%,EDNF%,EDLF%,EDKA%,  EDJF%,EDFA%,EDEA%,EDCA%,EDAF%,ECQM%,ECQH%,ECPF%,ECNF%,ECMA%,ECKA%,ECJF%,ECFA%,ECCA%,EBQM%,DGQM%,DGQH%,DGPF%,DGLF%,DGKA%,DGFA%,DGCA%,DGAF% |  |
| Stroke | G45%, G46%, I63% , I64%, I651, I66%, I67%, I68%, R47%, | EAFA%,EANF%,EAQH%,EAQM%,EBNF%,EBQH% |  |
| Valvular heart disease | I05x, I080x, I081x, I083x, I342x, Z952x, Z953x, Z954x I350x, I340x, I351x, I352x, I330x, I361x, I060x, I341x, Q231x, I062x, I348x, I371x', I339x, I38x, I398x, I088x, I089x', I349x, I358x, I391x, Q224x, Q230x | YYYY108, YYYY118, DBMA011, DGKA025, DGKA011, DBKA011, DBQM001, DBKA006, YYYY062, DBMA002, DBMA003, DZQJ002, DBLF001, DBKA011, DBKA009 |  |
| Hematologic disorders | D45,D75,D750,D751,D758,D759,D473 |  |  |
| Eclampsy | O10%,O11,O14%,O15% |  |  |
| Retinopathy | H34% |  |  |
| Lipid-lowering drug |  |  | C10AA, C10AB, C10AC01, C10AC02, C10BA02, C10BA05, C10AX, C10BX03 |
| Arterial hypertension | I10, I11, I15 |  | C02, C03, C07-C09 |
| Diabetes | E10x, E11x, E12x, E13x, E14x |  | A10A, A10B |
| Renal disorders | I120, I131, N032–N037, N052–N057, N18x, N19x, N250, Z490–Z492, Z940, Z992 |  |  |
| Antiulcer agent |  |  | A2B |
| Anti-platelet inhibitors |  |  | B01AC |
| Hematologic or immune diseases | D50 to D89 |  |  |
| NSAID |  |  | M01A, including M01AX02 (niflumic acid) and M01AX17 (nimesulide) but none of the other drugs labelled M01AX |
| Previous Bleeding | I312, I60-I62, I982, J942, K226, K252, K262, K270, K272, K280, K282, K290, K625, K661, K920-K922, M250, N939, R040-R043, R31, R58, S064-S066 |  |  |

**S3** **Table. Distribution of cardiovascular risk factors among patients treated for primary prevention with risk factors**

|  | **Monotherapy** | | |  | **Dual therapy** | | |
| --- | --- | --- | --- | --- | --- | --- | --- |
| **Characteristics** | **Aspirin Low**  **(≤100 mg)** | **Aspirin High**  **(> 100 - 325 mg)** | **Clopidogrel** |  | **ASA**  **+ CLOPIDOGREL** | **ASA**  **+ PRASUGREL** | **ASA**  **+ TICAGRELOR** |
|  | **N = 18 007** | **N = 3 393** | **N = 1 290** |  | **N = 390** | **N = 43** | **N = 56** |
| 1 risk factor | 9540 (53.0) | 2141 (63.1) | 757 (58.7) |  | 228 (58.5) | 27 (62.8) | 34 (60.7) |
| Diabetes mellitus | 1542 (6.65) | 181 (0.78) | 73 (0.31) |  | 9 (0.04) | 3 (0.01) | - |
| Arterial hypertension | 3398 (14.6) | 782 (3.37) | 268 (1.15) |  | 53 (0.23) | 2 (0.01) | - |
| Lipid-lowering drug | 4492 (19.4) | 1164 (5.02) | 409 (1.76) |  | 165 (0.71) | 22 (0.09) | 34 (0.15) |
| Renal disorders | 108 (0.47) | 14 (0.06) | 7 (0.03) |  | 1 (0.00) | - | - |
| 2 risk factors | 6427(35.7) | 1000(29.5) | 412(31.94) |  | 121(31.0) | 11(25.6) | 15(26.8) |
| Arterial hypertension / Diabetes mellitus | 735 (3.17) | 107 (0.46) | 57 (0.25) |  | 8 (0.03) | - | 2 (0.01) |
| Arterial hypertension / Lipid-lowering drug | 2223 (9.58) | 606 (2.61) | 227 (0.98) |  | 74 (0.32) | 5 (0.02) | 6 (0.03) |
| Arterial hypertension / Renal disorders | 161 (0.69) | 46 (0.20) | 10 (0.04) |  | 3 (0.01) | - | 2 (0.01) |
| Lipid-lowering drug / Renal disorders | 40 (0.17) | 6 (0.03) | 1 (0.00) |  | 4 (0.02) | 1 (0.00) | - |
| Lipid-lowering drug / Diabetes mellitus | 3258 (14.0) | 234 (1.01) | 117 (0.50) |  | 32 (0.14) | 5 (0.02) | 5 (0.02) |
| Renal disorders / Diabetes mellitus | 10 (0.04) | 1 (0.00) | - |  | - | - | - |
| At least 3 risk factors | 2040 (11.3) | 252 (7.43) | 121 (9.38) |  | 41 (10.51) | 5 (11.6) | 7 (12.5) |

**S4 Table. Bleeding site according to antiplatelet therapy**

|  | Monotherapy | | |  | Association (ASA +) | | |
| --- | --- | --- | --- | --- | --- | --- | --- |
| Characteristics | ASA Low | ASA High | Clopidogrel |  | Clopidogrel | Prasugrel | Ticagrelor |
|  | N = 45 881 | N = 13 702 | N = 4 017 |  | N =2 852 | N = 1 742 | N = 1 717 |
| Intracranial haemorrhage | 50 (0.11%) | 25 (0.18%) | 1 (0.02%) |  | 2 (0.07%) | 1 (0.06%) | 2 (0.12%) |
| Extra/subdural | 19 | 10 | 1 |  | 1 | 0 | 0 |
| Intracerebral | 26 | 13 | 0 |  | 1 | 1 | 2 |
| Other | 5 | 2 | 0 |  | 0 | 0 | 0 |
| Gastrointestinal (GI) bleeding | 59 (0.13%) | 22 (0.16%) | 5 (0.13%) |  | 7 (0.24%) | 7 (0.40%) | 6 (0.35%) |
| Upper GI | 41 | 17 | 3 |  | 5 | 2 | 3 |
| Lower GI | 18 | 5 | 2 |  | 2 | 5 | 3 |
| Other major bleeding | 37 (0.08%) | 6 (0.04%) | 7 (0.17%) |  | 6 (0.21%) | 3 (0.17%) | 4 (0.23%) |
| Hematuria / genitourinary | 14 | 2 | 3 |  | 1 | 1 | 2 |
| Oropharynx | 8 | 2 | 2 |  | 3 | 2 | 2 |
| Muscle (include soft tissue) | 4 | 1 | 1 |  | 1 | 0 | 0 |
| Other | 9 | 1 | 1 |  | 1 | 0 | 0 |
| All | 146 (0.32%) | 53 (0.39%) | 13 (0.32%) |  | 15 (0.53%) | 11 (0.63%) | 12 (0.70%) |

ASA denotes acetylsalicylic acid, Low (≤ 100 mg), High (> 100 - 325 mg)

**S5 Table. Values of crude incidence rates (per 10,000 person-months) of major bleeding according to antiplatelet regimen and time period**

**Panel A Monotherapy**

| Monotherapy | | | No of events | Person months | Incidence rate (95% CI) |
| --- | --- | --- | --- | --- | --- |
| 0 – 6 months | ICH | ASA Low | 19 | 187 440 | 1.01 (0.65 - 1.59) |
|  |  | ASA High | 17 | 50 266 | 3.38 (2.10 - 5.44) |
|  |  | Clopidogrel | 1 | 15 898 | 0.63 (0.09 - 4.47) |
|  | GI Bleeding | ASA Low | 28 | 187 440 | 1.49 (1.03 - 2.16) |
|  |  | ASA High | 13 | 50 266 | 2.59 (1.50 - 4.45) |
|  |  | Clopidogrel | 0 | 15 898 | 0.00 (. - .) |
|  | OT Bleeding | ASA Low | 13 | 187 440 | 0.69 (0.40 - 1.19) |
|  |  | ASA High | 3 | 50 266 | 0.60 (0.19 - 1.85) |
|  |  | Clopidogrel | 1 | 15 898 | 0.63 (0.09 - 4.47) |
|  | All Bleeding | ASA Low | 60 | 187 440 | 3.2 (2.49 - 4.12) |
|  |  | ASA High | 33 | 50 266 | 6.57 (4.67 - 9.23) |
|  |  | Clopidogrel | 2 | 15 898 | 1.26 ( 0.31 - 5.03) |
| 6 – 12 months | ICH | ASA Low | 15 | 121 233 | 1.24 (0.75 - 2.05) |
|  |  | ASA High | 4 | 27 975 | 1.43 (0.54 - 3.81) |
|  |  | Clopidogrel | 0 | 10 396 | 0.00 (. - .) |
|  | GI Bleeding | ASA Low | 5 | 121 233 | 0.41 (0.17 - 0.99) |
|  |  | ASA High | 5 | 27 975 | 1.79 (0.74 - 4.29) |
|  |  | Clopidogrel | 4 | 10 396 | 3.85 (1.44 - 10.25) |
|  | OT Bleeding | ASA Low | 9 | 121 233 | 0.74 (0.39 - 1.43) |
|  |  | ASA High | 1 | 27 975 | 0.36 (0.05 - 2.54) |
|  |  | Clopidogrel | 2 | 10 396 | 1.92 (0.48 - 7.69) |
|  | All Bleeding | ASA Low | 29 | 121 233 | 2.39 (1.66 - 3.44) |
|  |  | ASA High | 10 | 27 975 | 3.57 (1.92 - 6.64) |
|  |  | Clopidogrel | 6 | 10 396 | 5.77 (2.59 - 12.85) |
| > 12 months | ICH | ASA Low | 16 | 191 596 | 0.84 (0.51 - 1.36) |
|  |  | ASA High | 4 | 37 799 | 1.06 (0.40 - 2.82) |
|  |  | Clopidogrel | 0 | 16 823 | 0.00 (. - .) |
|  | GI Bleeding | ASA Low | 26 | 191 596 | 1.36 (0.92 - 1.99) |
|  |  | ASA High | 4 | 37 799 | 1.06 (0.40 - 2.82) |
|  |  | Clopidogrel | 1 | 16 823 | 0.59 (0.08 - 4.22) |
|  | OT Bleeding | ASA Low | 15 | 191 596 | 0.78 (0.47 - 1.30) |
|  |  | ASA High | 2 | 37 799 | 0.53 (0.13 - 2.12) |
|  |  | Clopidogrel | 4 | 16 823 | 2.38 (0.89 - 6.34) |
|  | All Bleeding | ASA Low | 57 | 191 596 | 2.98 (2.29 - 3.86) |
|  |  | ASA High | 10 | 37 799 | 2.65 (1.43 - 4.92) |
|  |  | Clopidogrel | 5 | 16 823 | 2.97 (1.24 - 7.14) |

ASA denotes acetylsalicylic acid. ICH denotes intracranial hemorrhage, GI gastrointestinal and OT other major bleeding.

**Panel B Dual antiplatelet regimen**

| Dual antiplatelet regimen | | | No of events | Person months | Incidence rate (95% CI) |
| --- | --- | --- | --- | --- | --- |
| 0 – 3 months | ICH | ASA + Clopidogrel | 0 | 6 635 | 0 ( - ) |
|  |  | ASA + Prasugrel | 1 | 4 852 | 2.06 (0.29 - 14.63) |
|  |  | ASA + Ticagrelor | 1 | 4 437 | 2.25 (0.32 - 16.00) |
|  | GI Bleeding | ASA + Clopidogrel | 3 | 6 635 | 4.52 (1.46 - 14.02) |
|  |  | ASA + Prasugrel | 5 | 4 852 | 10.31 (4.29 - 24.76) |
|  |  | ASA + Ticagrelor | 1 | 4 437 | 2.25 (0.32 - 16.00) |
|  | OT Bleeding | ASA + Clopidogrel | 3 | 6 635 | 4.52 (1.46 - 14.02) |
|  |  | ASA + Prasugrel | 1 | 4 852 | 2.06 (0.29 - 14.63) |
|  |  | ASA + Ticagrelor | 0 | 4 437 | 0.00 (. - .) |
|  | All Bleeding | ASA + Clopidogrel | 6 | 6 635 | 9.04 (4.06 - 20.13) |
|  |  | ASA + Prasugrel | 7 | 4 852 | 14.43 (6.88 - 30.26) |
|  |  | ASA + Ticagrelor | 2 | 4 437 | 4.51 (1.13 - 18.02) |
| 3 – 6 months | ICH | ASA + Clopidogrel | 1 | 4 611 | 2.17 (0.31 - 15.40) |
|  |  | ASA + Prasugrel | 0 | 4 056 | 0.00 ( - ) |
|  |  | ASA + Ticagrelor | 0 | 3 245 | 0.00 ( - ) |
|  | GI Bleeding | ASA + Clopidogrel | 1 | 4 611 | 2.17 (0.31 - 15.40) |
|  |  | ASA + Prasugrel | 0 | 4 056 | 0.00 (. - .) |
|  |  | ASA + Ticagrelor | 2 | 3 245 | 6.16 (1.54 - 24.65) |
|  | OT Bleeding | ASA + Clopidogrel | 2 | 4 611 | 4.34 (1.08 - 17.34) |
|  |  | ASA + Prasugrel | 1 | 4 056 | 2.47 (0.35 - 17.50) |
|  |  | ASA + Ticagrelor | 2 | 3 245 | 6.16 (1.54 - 24.65) |
|  | All Bleeding | ASA + Clopidogrel | 4 | 4 611 | 8.67 (3.26 - 23.11) |
|  |  | ASA + Prasugrel | 1 | 4 056 | 2.47 (0.35 - 17.50) |
|  |  | ASA + Ticagrelor | 4 | 3 245 | 12.33 (4.63 - 32.85) |
| > 6 months | ICH | ASA + Clopidogrel | 1 | 8 735 | 1.14 (0.16 - 8.13) |
|  |  | ASA + Prasugrel | 0 | 7 295 | 0.00 (. - .) |
|  |  | ASA + Ticagrelor | 1 | 4 964 | 2.01 (0.28 - 14.30) |
|  | GI Bleeding | ASA + Clopidogrel | 3 | 8 735 | 3.43 (1.11 - 10.65) |
|  |  | ASA + Prasugrel | 2 | 7 295 | 2.74 (0.69 - 10.96) |
|  |  | ASA + Ticagrelor | 3 | 4 964 | 6.04 (1.95 - 18.74) |
|  | OT Bleeding | ASA + Clopidogrel | 1 | 8 735 | 1.14 (0.16 - 8.13) |
|  |  | ASA + Prasugrel | 1 | 7 295 | 1.37 (0.19 - 9.73) |
|  |  | ASA + Ticagrelor | 2 | 4 964 | 4.03 (1.01 - 16.11) |
|  | All Bleeding | ASA + Clopidogrel | 5 | 8 735 | 5.72 (2.38 - 13.75) |
|  |  | ASA + Prasugrel | 3 | 7 295 | 4.11 (1.33 - 12.75) |
|  |  | ASA + Ticagrelor | 6 | 4 964 | 12.09 (5.43 - 26.90) |

ASA denotes acetylsalicylic acid. ICH denotes intracranial hemorrhage, GI gastrointestinal and OT other major bleeding.

**S6 Table. Monotherapy - Baseline characteristics in unweighted (baseline) and weighted population**

**Panel A: Primary prevention without risk factors**

|  | ASA Low | ASA High | Clopidogrel | Unweighted | Weighted |
| --- | --- | --- | --- | --- | --- |
|  | N = 15 561 | N = 3 499 | N =701 | SD | |
| Age, mean year | 55 | 58 | 65 | 0.148 | 0.024 |
| Gender, female | 68.2 | 55.2 | 49.2 | 0.269 | 0.019 |
| Chronic pulmonary disease | 8.35 | 8.55 | 14.7 | 0.007 | 0.006 |
| Cancer | 3.09 | 3.29 | 3.71 | 0.011 | 0.009 |

SD stands for Standardised difference

**Panel B: Primary prevention with risk factors**

|  | ASA Low | ASA High | Clopidogrel | Unweighted | Weighted |
| --- | --- | --- | --- | --- | --- |
|  | N =18 007 | N = 3 393 | N = 1 290 | SD | |
| Age, mean year | 69 | 69 | 70 | 0.061 | 0.034 |
| Gender, female | 52.3 | 51.6 | 51.4 | 0.014 | -.013 |
| Diabetes mellitus | 41.6 | 22.2 | 28.1 | -.425 | -.008 |
| Cerebrovascular disease | 2.6 | 18.0 | 5.7 | 0.522 | -.001 |
| Chronic pulmonary disease | 11.2 | 11.9 | 13.6 | 0.023 | -.002 |
| Cancer | 3.5 | 4.2 | 3.3 | 0.041 | 0.011 |
| Hypertension uncontrolled | 47.5 | 52.8 | 52.9 | 0.107 | 0.024 |
| Impaired renal | 3.1 | 3.3 | 3.0 | 0.012 | 0.012 |

SD stands for Standardised difference

**Panel C: Secondary prevention**

|  | ASA Low | ASA High | Clopidogrel | Unweighted | Weighted |
| --- | --- | --- | --- | --- | --- |
|  | N =12 313 | N = 6 810 | N = 2 026 | SD | |
| Age, mean year | 67 | 65 | 66 | -.069 | 0.008 |
| Gender, female | 43.7 | 47.5 | 38.7 | -.076 | 0.007 |
| Ischemic heart disease | 10.6 | 4.0 | 2.7 | -.255 | 0.038 |
| Diabetes mellitus | 18.0 | 10.3 | 18.0 | -.221 | 0.040 |
| Congestive heart failure | 4.0 | 2.0 | 1.3 | -.121 | 0.021 |
| Peripheral vascular disease | 9.6 | 7.6 | 25.6 | -.072 | 0.015 |
| Cerebrovascular disease | 7.9 | 64.1 | 14.0 | 1.448 | -.008 |
| Chronic pulmonary disease | 12.5 | 10.8 | 14.2 | -.051 | 0.007 |
| Hemiplegia | 1.4 | 14.7 | 3.75 | 0.501 | 0.001 |
| Cancer | 3.8 | 3.7 | 3.1 | -.006 | 0.005 |
| Hypertension uncontrolled | 35.8 | 38.0 | 32.4 | 0.046 | 0.027 |
| Previous history of stroke | 2.3 | 19.7 | 4.2 | 0.578 | -.011 |

SD stands for Standardised difference

**Non-author point of contact: info@indsante.fr**

**List of relevant data set names to request our minimal data set**

| DATAMART | TABLE | Label |
| --- | --- | --- |
| DCIR | ER_BIO_F | Biological tests |
| DCIR | ER_CAM_F | Medical procedures |
| DCIR | ER_ETE_F | Hospital data |
| DCIR | ER_PHA_F | Drugs data |
| DCIR | ER_PRS_F | Health expenditure |
| DCIR | IR_BEN_R | Patient data |
| PMSI | T_MCOaaA | Medical procedures |
| PMSI | T_MCOaaB | Hospital stays |
| PMSI | T_MCOaaC | Hospital stays diagnosis |
| PMSI | T_MCOaaCSTC | Outpatient consultations |
| PMSI | T_MCOaaD | Discharge associated diagnosis ICD-10 |
| PMSI | T_MCOaaE | Hospital data |
| PMSI | T_MCOaaFBSTC | Outpatient consultations expenditure |
| SACHA | Linkage Table | Linkage variables, type and date of major bleeding |
