# Supplementary material for: Major bleeding risk and mortality associated with antiplatelet drugs in real-world clinical practice. A prospective cohort study
Source: PLoS One. 2020 Aug 7;15(8):e0237022. doi: 10.1371/journal.pone.0237022 (PMC7413418; doi:10.1371/journal.pone.0237022)
Supplement: Attachment — Submitted filename: Response to Editor.docx [file pone.0237022.s004.docx]

**Editor Comments :**

**The authors responded to the reviewers comments but the relevant response was not incorporated in the appropriate section /discussion section except one line in limitation section.**

**Please incorporate the relevant part to the appropriate section of the manuscript.**

**Reviewer #1:**

**1. CAD is mentioned as one category but we know that there is a significant difference between stable CAD and ACS.**

The following paragraph has been added in the R2 revised version, lines 181 to 185.

"Out of 5,674 patients classified as having coronary artery disease as presumed indication for antiplatelet monotherapy, 1,026 had been hospitalized (out of them 705 suffered from ACS) and 4,048 had ambulatory care for angina pectoris identified through stress tests. Then 705 out of 5,674 patients (12.4%) suffered from ACS."

**2. We know that Effient is more potent and causes more bleeding than other antiplatelets medications. Is there a reason why the data did not show significant differences between dual antiplatelets regimens ?**

The following paragraph has been added in the R2 revised version, lines 292 to 294.

"We observed very few events in patients prescribed with dual regimens, keeping in mind only major bleeding were accounted for; given these low incidences we had a very low power to detect any difference."

**Reviewer #2:**

**3. Please define major bleeding, fatal vs. nonfatal bleeding as mentioned in the article. Is there a specific/standardized score that has been used? It would be a good idea to include various bleeding scores like BARC bleeding scale, GUSTO, etc.**

The following sentences have been added in the R2 revised version, lines 306 to 315.

"We analyzed a population exposed to antiplatelet agents, irrespective of the indication, then including a larger scope than coronary artery disease. This is the reason why we did not consider bleeding scores devoted to coronary artery disease. Moreover, we focused on major bleeding occurring after discharge when antiplatelet agents were prescribed in secondary prevention, that is after an hospitalization for an acute thrombotic event. We did not collected early major bleeding after medical or surgical procedures. All bleeding events were medically validated using pre-specified criteria which were somewhat consistent with the "severe or life-threatening' item from GUSTO. The systematic medical validation of all bleeding limited the overestimation of incidence rates of major bleeding [23]."

**5. Angers, Brest, Grenoble, Nantes and Rennes were the five cities from France from where the data was obtained. Is this data generalizable to French population vs. world in general.**

The following sentences have been added in the R2 revised version, lines 320 to 325.

"The design and linkage to Health Insurance database allowed to state the study was exhaustive within five well-defined areas around five large French cities. But translating the result to French population is more hazardous. However, we could hypothesize that the reasons why those patients were referred to emergency department for major bleeding should not be different in other French regions. Of note, racial data cannot be collected in France. We hypothesized our population was mostly of Caucasian descent."
